# Role of Immunological Memory Cells as a Therapeutic Target in Multiple Sclerosis

**DOI:** 10.3390/brainsci7110148

**Published:** 2017-11-07

**Authors:** Tanima Bose

**Affiliations:** Institute of Human Genetics, University of Regensburg, Franz-Josef-Strauss-Allee 11, D-93053 Regensburg, Germany; tanimabose@gmail.com or tanima.bose@ur.de; Tel.: +49-941-944-5449; Fax: +49-941-944-5402

**Keywords:** multiple sclerosis, MS, central memory T cells, T_CM_, effector memory T cells, T_EM_, resident memory T cells, T_RM_

## Abstract

Pharmacological targeting of memory cells is an attractive treatment strategy in various autoimmune diseases, such as psoriasis and rheumatoid arthritis. Multiple sclerosis is the most common inflammatory disorder of the central nervous system, characterized by focal immune cell infiltration, activation of microglia and astrocytes, along with progressive damage to myelin sheaths, axons, and neurons. The current review begins with the identification of memory cell types in the previous literature and a recent description of the modulation of these cell types in T, B, and resident memory cells in the presence of different clinically approved multiple sclerosis drugs. Overall, this review paper tries to determine the potential of memory cells to act as a target for the current or newly-developed drugs.

## 1. Recent Insights into Inflammatory Neuronal Injury in Multiple Sclerosis

Multiple sclerosis is one of the most prominent demyelinating disorders, and makes a bridge between immune and neuronal systems by degenerating the neuronal myelin sheath through a series of inflammatory mechanisms. Scientists over the decades have attempted to investigate the exact immune mechanisms underlying the degeneration of the myelin sheath. 

The classification of multiple sclerosis is as clinically isolated syndrome (CIS), primary progressive multiple sclerosis (PPMS), secondary progressive multiple sclerosis (SPMS), and relapsing-remitting multiple sclerosis (RRMS), depending on the progression and relapses of the disease [1]. The roles of memory T or B cells are prominent in each of the different forms of the disease, but its role is more prominent in the relapsing forms, as explained later in detail. The interesting fact is that the multiple sclerosis drugs prescribed for various forms of multiple sclerosis have a major impact on the functionality and abundance of T and B memory cells. Memory cells by definition are a group of cells which have the experience of antigen recognition in a lifetime of T or B cells. They represent the distinctive features of the adaptive immune functionality, and their mode of action and phenotypic features are distinct depending on the cell types. Human memory T cells, B cells, and resident memory T cells are CD45RO^+^CD45RA^−^, IgD^+^CD27^+^, and CD69^+^CD103^+^, respectively (cell surface antigens). The origins and functions of the T and B cells are different, but both T and B cells have the same division of labor: plasma cells secreting antibodies in the B cell part does the job of protective memory, and effector memory T cells (T_EM_s) does the same function by migrating immediately to the inflamed peripheral tissue and displaying necessary effector functions. Memory B cells perform the function of proliferation and stimulation in response to antigenic stimulation, whereas central memory T cells (T_CM_s) do the same job by homing in the secondary lymphoid organs and readily transform into T_EM_s while encountering the antigens [2]. To support this function, T_CM_s express chemokine receptor CCR7 and the adhesion molecule L selectin (CD62L), allowing them to access the lymph nodes from blood, and T_EM_s express low levels of these two chemokine receptors, permitting them to approach peripheral tissue such as skin [3,4]. T_CM_s home to the lymph nodes and have a limited capacity to have effector functions until they are stimulated by the secondary responses, whereas T_EM_s home to peripheral tissue and rapidly produce effector cytokines upon antigenic stimulation. The effector cell type can give rise to the long-lasting tissue-resident memory T cells (T_RM_s) which might protect against multiple encounters of the similar group of pathogens, and which might help to develop vaccines or drugs in future [5]. 

## 2. Role of Memory T Cells in the Pathogenesis of Multiple Sclerosis

An earlier report from the group of Hedlund, G. et al. has shown that the sustained increase of CD4^+^ memory T cells in the cerebrospinal fluid of multiple sclerosis patients compared to the peripheral blood was a normal phenomenon [6]. A later report in the 1990s by the group of Zaffaroni, M. et al. observed the augmented conversion from naïve to memory cells in chronic-progressive multiple sclerosis [7]. Further, there is a definite trend of increase in memory CD45RO^+^CD4^+^ T cells and a decrease in naïve CD45RA^+^ T cells in the peripheral blood of multiple sclerosis patients. Additionally, there is a significant elevation of CD4/CD8 ratio [8]. In parallel, the role of memory cells in identifying the myelin basic protein (MBP) or myelin antigen-specific T cells was continuously explored in several publications. Most of the myelin-reactive T cells were shown to exist in the memory T cell subset [9]. Memory T cells are activated and proliferated even with the lack of CD28 co-stimulation [10,11]. Thus, this kind of co-stimulation blockade is not an effective strategy to prevent the MS responses. Besides CD28, later study initiated the chance of Inducible COStimulator (ICOS)-co-stimulation as an effective target for the autoimmune demyelinating disorder [12,13,14]. As mentioned, CD4^+^CD28^−^ cells have the full potential to proliferate in the central nervous system—a site which is devoid of any professional antigen-presenting cells [15]. During this period, there was also a search to determine if any cytokine has the potential to enhance the effector function of memory cells upon adoptive transfer. It was indeed possible to find that the transforming growth factor-beta has the efficiency to increase the memory phenotype of the cultured cells and effector function of the cells upon adoptive transfer into an experimental autoimmune encephalomyelitis animal model [16]. An enhanced expression of CD45RO^+^ memory T cells and decreased expression of CD45RA^+^ naïve T cells while immunophenotyping the peripheral blood from the patients of another form of neurodegeneration (Parkinson’s Disease) was also observed around this time [17]. After the establishment of the role of memory T cells as one of the major culprits, there was a continuous trial to determine which memory subset is important in case of the presence of disease or application of the drug. Some of the examples from this investigation are the following: In a transcriptomic study, fingolimod increases the effect of CCR7^−^ T_EM_s in the peripheral blood of the patients [18,19,20,21]. On the other hand, another important drug for this disease, dimethyl fumarate (methyl ester of fumaric acid), was shown to lower the proportion of circulating T_CM_ and T_EM_ in compared to naïve T cells [22]. Further, there was a decreased presence of Th1 CD4^+^ cells, increase in the abundance of Th2 CD8^+^ cells, and an unaltered presence of Th17^+^ cells in the presence of this drug [23]. Interestingly, another clinically approved drug, natalizumab (monoclonal antibody targeting adhesion molecule α4-integrin), increased the IFN-γ and IL17A cytokines secreted by CD4^+^ memory T cells and reduced the CD49d^+^ Treg cells more than the Th1 or Th17 cells [24]. In contrast, a later study showed unchanged memory, naïve, or effector T cells with the affected B cell population [25]. In the presence of other two approved drugs *viz* interferon beta (glycoprotein) and glatiramer acetate (immunomodulator), there was a beneficial decline of T_CM_s and an increase of naïve cells [26]. In a recent paper, there was an attempt to explain the association between MS, viral infection, and MS-drugs (fingolimod and natalizumab). They pointed out Th1/Th17 central memory cells can be targeted to protect from both the MS-induced relapses and virus-induced encephalomyelitis [27]. The investigation also found that memory cells have a favorable phenotype compared to the naïve cells to breach the blood–brain barrier. The reason being was the invadosome-like protrusions in them were 2–3 fold increased compared to the crawling naïve T cells that helped them to cross long distances (150 µm) on endothelial tight junctions before crossing the blood–brain barrier [28,29]. As the functions and origins of T_CM_ and T_EM_ differ, the modulation of these populations either in the lymph node or periphery in the presence of several MS drugs can also have an aftermath effect on relapses after exposure to the drugs. 

## 3. Role of Memory B Cells in the Pathogenesis of Multiple Sclerosis

The depletion of CD19^+^CD27^+^ B memory cells in the presence of natalizumab and the long-term persistence of this status in the presence of other depleting factors like CD52 and CD20 strengthened the importance of B memory cells in this autoimmune demyelinating disorder [30]. Along with this line, the investigations supported the depletion of memory B cells in presence of other MS drugs, as observed in case of memory T cells. Exploring different kinds of memory cells also resolves the underlying mechanism of action of the drugs. For example, the therapeutic mode of action of dimethyl fumarate (DMF) in treating relapsing-remitting multiple sclerosis is still not properly understood. In a recent paper, memory B cells in circulating mature/differentiated B cell type was significantly diminished while treating with this drug. The DMF-mediated decrease leads to the reduction of the pro-inflammatory signals (GM-CSF, IL-6, TNF-α) compounded with reduced phosphorylation of STAT5/6 and NF-κB in surviving B cells [31]. An earlier report mentioned that this drug increased the amount of B cells with regulatory capacity (IL-10 producing B cells) [32]. Fingolimod used for treating relapsing-remitting multiple sclerosis was shown to have broad effects on the increase/decrease of the cell populations similar to DMF. It increases the naïve to memory cell phenotype, modulates the circulatory B cells with an abundance of regulatory capacity and an increase of anti-inflammatory cytokines [33]. Another first-line disease-modifying drug, interferon-beta (IFN-β), has both anti-inflammatory properties and can effectively target the memory B cells [34]. To determine whether a single dose of the drug is sufficient to eradicate the disease-causing cell subsets, it is elucidated that a single dose of rituximab did not eliminate the IgG memory B cells and might facilitate the presence of auto-reactive immune cells [35]. Along with memory B cells, the exploration of CD40 co-stimulation helped in identifying the mechanistic pathway of the currently existing drugs. To support that, CD40-mediated elevation in pP65 (NF-κB) level was observed in the naïve and memory cells from the relapsing-remitting and progressive multiple sclerosis patients compared to the control subjects [36]. Further, the combination therapy of IFN-β-1a (Avonex) and mycophenolate mofetil (Cellcept) and glatiramer acetate leads to the modulation of hyperphosphorylation of P65 in B cells [36]. There was an intention to search for the signaling molecule responsible for the propagation of granulocyte macrophage colony-stimulating factor (GM-CSF) memory B cells, and it was found that the signal transducer and activator of transcription 5/6 (STAT5/6)-regulated mechanistic pathway is upregulated in untreated MS patients, and this also reciprocally regulates the IL-10 secretion [37]. It is also interesting to observe how different external factors (e.g., Epstein-Barr virus, EBV) modulate the self- and poly-reactivity of memory B cells. In the case of EBV infection, memory cells have evolved to have less self-reactivity which gives the virus an opportunity to propagate more in B-memory cell type in contrast to others [38].

Table 1 explains a brief overview of the relationship between clinically-approved MS drugs and modulation of memory cell types.

## 4. Role of Resident Memory Cells in Mediating Demyelinating Disorders

T_RM_s are the new bunch of memory cells having different transcriptional programs than effector and central memory T cells. They are mostly present in the barrier tissues like the skin and gut. Among the two populations of memory T cells (recirculating and resident) present in the peripheral tissue, residency of the cells in the case of both CD4^+^ and CD8^+^ are determined by CD103 expression [44,45]. Other than CD103, the prominent activation marker used to identify the T_RM_s is CD69 [44,46]. In certain tissues (e.g., skin and intestinal epithelium), there is no requirement of antigen presentation for the CD103^+^CD8^+^ T_RM_s formation as a consequence of TGF-β signaling [46,47]. Along with the barrier tissues, there are recent reports of the presence of T_RM_s in other non-barrier tissues like kidney and joint inflammation. T_RM_s protect the barrier tissue against environmental pathogens, but a recent report observed that T_RM_ is generated in response to a topically-applied allergen. In a recent publication, T_CM_ was shown to match T_RM_ in terms of their functions, *viz* being stimulated by the secondary responses [48]. The T_RM_s were shown to present in the brain, evading the blood–brain barrier [49]. In this kind of CD103+CD8+ expression, the local antigen stimulation for CD103^+^ is necessary. T_RM_s were present in the brain tissue after the in vitro infection with vesicular stomatitis virus, and the effector population here was CD8^+^CD103^+^ type, but the factor required for the continuous stimulation of T cells is still unclear. At the transcriptional level, brain T_RM_ resembles well with the skin, gut, and lungs but they are transcriptionally distinct from central and effector memory population [49,50]. There is still a lack of evidence as to whether these kinds of memory cells are indeed present in the brain. There is a recent report that supports the presence of CD8^+^ T_RM_s in MS patients. In this report, relapsing-remitting and chronic forms of the disease were mediated by the tissue-resident CD8^+^ lymphocytes, and the acute form of the disease was regulated by the effector memory population residing in the meninges and perivascular space [51].

## 5. Novel Therapies Targeting Memory Cells with a Future in Clinical Development

The most important knowledge that the modulation of memory cells brings to us is the modification of the MS patients’ immune profiles while taking the clinically-approved drugs. The immune-modulating mechanism in the case of both T or B cells is the elevation of naïve immune cells compared to the memory cells and the shift towards the anti-inflammatory paradigm, both of which ensure the elimination of auto-aggressive immune cells. With the increasing knowledge, the final goal will be to use different immunomodulators which may prevent the relapsing of MS. One such example of the new class of modulator is VitD3, the application of which in vitro in the peripheral blood mononuclear cells can abrogate the proportion of effector memory T cells and enhance the abundance of naïve cells [52]. Further investigations in this direction may yield innovative treatment either with the existing approved drugs, or in combination with other new classes of immunomodulators. 

## Figures and Tables

**Table 1:** The effect of multiple sclerosis (MS) drugs on different kinds of memory cells.

MS Drugs	Memory T Cells	Memory B Cells
Fingolimod	Increase T_CM_s [39]	Decreased [40]
Dimethyl fumarate	Decrease of T_EM_ and T_CM_s [41]	Decreased [32]
Natalizumab	Unchanged [25]	Increased [25]
Interferon-β	Decrease of T_CM_s [26]	Decreased [34]
Glatiramer acetate	Decrease of T_CM_s [26]	Decreased [42]
Teriflunomide	Not known	Decreased [43]
Dalfampridine	Not known	Not known
